# Self-supervised learning of accelerometer data provides new insights for sleep and its association with mortality

**DOI:** 10.1038/s41746-024-01065-0

**Published:** 2024-06-30

**Authors:** Hang Yuan, Tatiana Plekhanova, Rosemary Walmsley, Amy C. Reynolds, Kathleen J. Maddison, Maja Bucan, Philip Gehrman, Alex Rowlands, David W. Ray, Derrick Bennett, Joanne McVeigh, Leon Straker, Peter Eastwood, Simon D. Kyle, Aiden Doherty

**Affiliations:** 1 Nuffield Department of Population Health, University of Oxford, Oxford, UK; 2 Big Data Institute, Li Ka Shing Centre for Health Information and Discovery, University of Oxford, Oxford, UK; 3 Diabetes Research Centre, University of Leicester, Leicester, UK; 4 College of Medicine and Public Health, Flinders University, Adelaide, SA Australia; 5 Centre of Sleep Science, School of Human Sciences, University of Western Australia, Perth, WA Australia; 6 West Australian Sleep Disorders Research Institute, Department of Pulmonary Physiology, Sir Charles Gairdner Hospital, Nedlands, WA Australia; 7 Department of Genetics, University of Pennsylvania, Philadelphia, PA USA; 8 Department of Psychiatry, University of Pennsylvania, Philadelphia, PA USA; 9 NIHR Leicester Biomedical Research Centre, University of Leicester, Leicester, UK; 10 NIHR Oxford Biomedical Research Centre, John Radcliffe Hospital, Oxford, UK; 11 Oxford Centre for Diabetes, Endocrinology and Metabolism, Oxford Kavli Centre for Nanoscience Discovery, University of Oxford, Oxford, UK; 12 Medical Research Council Population Health Research Unit, University of Oxford, Oxford, UK; 13 Curtin School of Allied Health, Curtin University, Perth, WA Australia; 14 Health Futures Institute, Murdoch University, Murdoch, WA Australia; 15 Sir Jules Thorn Sleep & Circadian Neuroscience Institute, Nuffield Department of Clinical Neurosciences, University of Oxford, Oxford, UK

**Keywords:** Epidemiology, Translational research

## Abstract

Sleep is essential to life. Accurate measurement and classification of sleep/wake and sleep stages is important in clinical studies for sleep disorder diagnoses and in the interpretation of data from consumer devices for monitoring physical and mental well-being. Existing non-polysomnography sleep classification techniques mainly rely on heuristic methods developed in relatively small cohorts. Thus, we aimed to establish the accuracy of wrist-worn accelerometers for sleep stage classification and subsequently describe the association between sleep duration and efficiency (proportion of total time asleep when in bed) with mortality outcomes. We developed a self-supervised deep neural network for sleep stage classification using concurrent laboratory-based polysomnography and accelerometry. After exclusion, 1113 participant nights of data were used for training. The difference between polysomnography and the model classifications on the external validation was 48.2 min (95% limits of agreement (LoA): −50.3 to 146.8 min) for total sleep duration, −17.1 min for REM duration (95% LoA: −56.7 to 91.0 min) and 31.1 min (95% LoA: −67.3 to 129.5 min) for NREM duration. The sleep classifier was deployed in the UK Biobank with ~100,000 participants to study the association of sleep duration and sleep efficiency with all-cause mortality. Among 66,262 UK Biobank participants, 1644 mortality events were observed. Short sleepers (<6 h) had a higher risk of mortality compared to participants with normal sleep duration 6–7.9 h, regardless of whether they had low sleep efficiency (Hazard ratios (HRs): 1.36; 95% confidence intervals (CIs): 1.18 to 1.58) or high sleep efficiency (HRs: 1.29; 95% CIs: 1.04–1.61). Deep-learning-based sleep classification using accelerometers has a fair to moderate agreement with polysomnography. Our findings suggest that having short overnight sleep confers mortality risk irrespective of sleep continuity.

## Introduction

Sleep is essential to life and is structurally complex. Humans spend approximately one third of their lives asleep, yet sleep is hard to assess in free-living environments^1^. Our understanding of how sleep is associated with health and morbidity primarily draws on studies that use self-report sleep diaries, which capture the subjective experience^2^. However, sleep diaries have a low correlation with objective device-measured sleep parameters^3,4^. The accepted standard for sleep measurement is laboratory-based polysomnography, which monitors sleep using a range of physical and physiological signals. However, polysomnography is not feasible for use at scale due to its high cost and technical complexity. Instead, wrist-worn accelerometers are more viable to deploy in large-scale epidemiological studies because of their portability and low user burden.

Despite the popularity of sleep monitoring in consumer and research-grade wrist-worn devices, sleep assessment algorithms are frequently proprietary and validated in small populations, making their measurement validity unclear^5–8^. Methods for Sleep classification (i.e. defining periods of wake, NREM and REM sleep) primarily rely on hand-crafted spatiotemporal features such as device angle, which may not make full use of all the information in the signals. Hence, data-driven methods like deep learning could be advantageous. Furthermore, existing actigraphy-based sleep studies on large health datasets have only focused on the differentiation between sleep and wakefulness^4,9–11^ without evaluating variations in the stages of sleep.

We therefore set out to: (1) develop and internally validate an open-source novel deep learning method to infer sleep stages from wrist-worn accelerometers, (2) externally validate our proposed algorithm together with existing sleep staging benchmarks and (3) investigate the association between device-measured overnight sleep duration and efficiency with all-cause mortality.

## Results

**Fig. 1:**
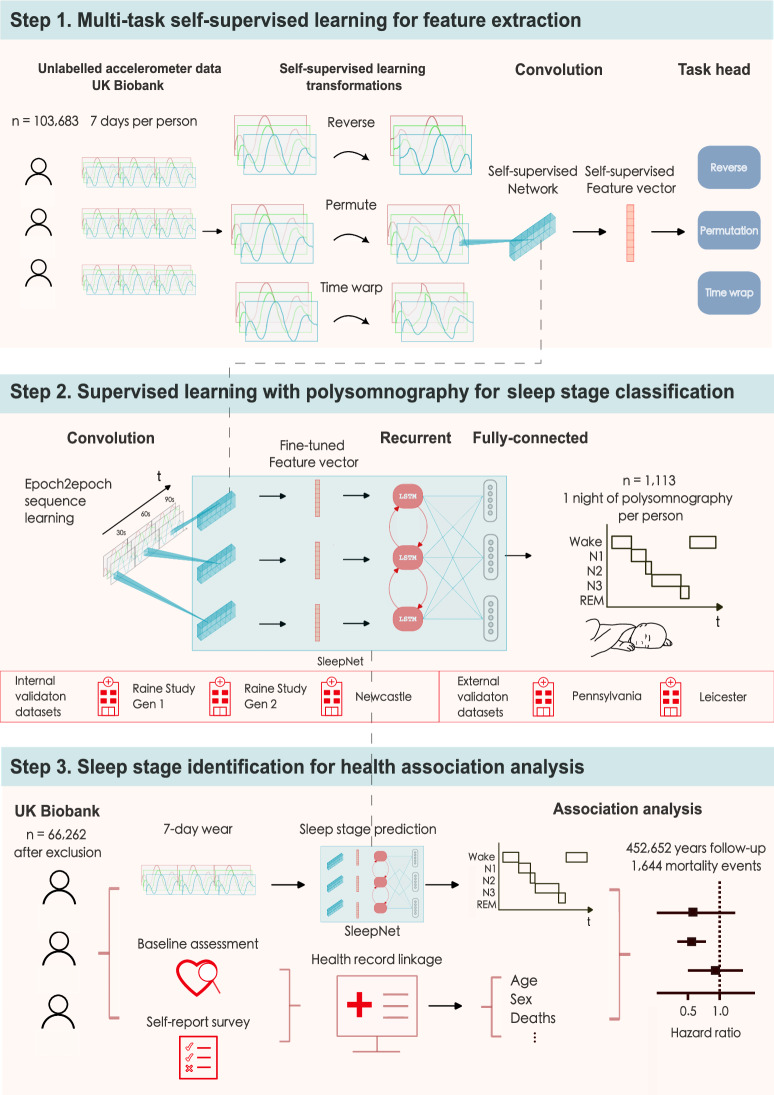
The SleepNet development pipeline. 1. We use multi-task self-supervised learning to obtain a feature extractor by learning from 700,000 person-days of tri-axial accelerometry data in the UK Biobank. 2. The pre-trained feature extractor was then fine-tuned with a deep recurrent network to train a sleep-stage classifier using polysomnography as the ground truth. 3. We deploy the sleep prediction model on the UK Biobank and investigate the association between device-measured sleep and mortality outcomes.

In our multicentre cohort study, we developed and tested a sleep staging model for accelerometers (SleepNet) using a self-supervised deep recurrent neural network. We designed the model to classify each 30-s window of accelerometry data into one of the three sleep stages, wake, rapid-eye-movement sleep (REM) and non-rapid-eye movement sleep (NREM). Figure 1 illustrates the three main steps in our study: (1) feature extraction from unlabelled free-living data, (2) sleep staging model development and (3) face validity assessment and health association analysis using the machine learning-estimated sleep parameters.

### Comparison to polysomnography

After preprocessing, 1113 participants were included in the internal validation and 53 participants were included in the external validation. Our proposed deep recurrent neural network (SleepNet) pre-trained with self-supervision achieved the best performance when compared with other baseline models that used hand-crafted features (Supplementary Table 6).

**Fig. 2:**
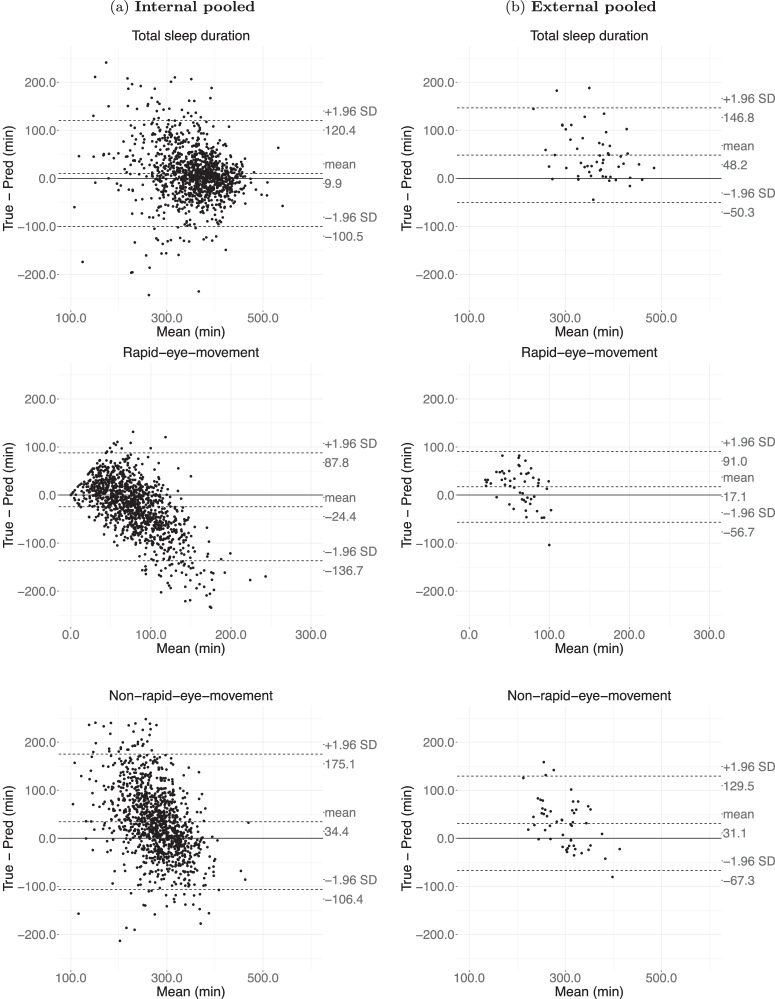
Agreement assessment via Bland-Atman plot for total sleep duration, rapid eye movement sleep (REM) duration and non-rapid eye movement sleep (NREM) duration on internal and external validation. **a** the agreement for the internal validation; **b** the agreement for the external validation. The internal validation consists of 1113 polysomnography nights from the Raine Study and the Newcastle cohort, whereas the external validation consists of 53 polysomnography nights from the Leicester and Pennsylvania cohorts.

On the internal validation, SleepNet had a mean bias of 9.9 min (95% limits of agreement (LoA): −100.5–120.4 min) for total sleep duration, −24.4 min (95% LoA: −136.7–87.8 min) for REM duration and 34.4 min (95% LoA: −106.4–175.1 min) for NREM duration (Fig. 2). In comparison, on the external validation, the mean bias was 48.2 min (95% LoA: −50.3–146.8 min) for total sleep duration, −17.1 min (95% LoA: −56.7–91.0 min) for REM duration and 31.1 min (95% LoA: −67.3–129.5 min) for NREM duration. Overall, our model tends to underestimate REM and short sleep and overestimate NREM and long sleep. Supplementary Figs. 5–10 depict the agreement assessments for other sleep parameters on the individual cohorts.

The subject-wise performance for both the internal and external validation using the pre-trained SleepNet is shown in Supplementary Table 7. On the pooled internal validation, our model obtained an F1 of 0.75 ± 0.1 in the two-class setting (sleep/wake) and an F1 of 0.57 ± 0.11 in the three-class setting (wake/REM/NREM). The agreement decreased slightly on the external validation with an F1 of 0.66 ± 0.12 in the two-class setting (sleep/wake) and an F1 of 0.49 ± 0.10 in the three-class setting (wake/REM/NREM). In the Newcastle cohort, for the sleep/wake classification, sensitivity decreased and specificity increased in participants with sleep disorders. No obvious difference was observed in both Raine Gen1 and Gen2 cohorts when the participants were stratified by sex, BMI, AHI and sleep disorder conditions. (Supplementary Tables 8–10).

To classify any given window in an epoch-by-epoch fashion, the SleepNet achieved a Kappa score of 0.39 on the internal validation set and a Kappa score of 0.32 on the external validation set in the three-class setting (Supplementary Fig. 11). Cohort-specific confusion matrices can be found in Supplementary Figs. 12–15. Supplementary Fig. 16 visualises a one-night sample actigram, its ground-truth polysomnography labels and SleepNet predictions. We used SleepNet to generate all the sleep parameters for the rest of the paper.

### Face validity in the UK Biobank

Before deploying the SleepNet on the UK Biobank, we excluded participants with unusable accelerometer data and participants with missing covariates in the descriptive analysis. We further excluded participants with any prior hospitalisation for cardiovascular disease or cancer in the association analysis (Supplementary Fig. 17). In sum, 66,262 participants were included in the final analysis.

Table 1 describes the variations in overnight sleep duration, REM and NREM durations and sleep efficiency across population subgroups in the UK Biobank. Older participants generally slept longer with higher sleep efficiency. Females had a longer overnight sleep duration, REM and NREM durations. Participants with better self-rated health had longer sleep duration and higher sleep efficiency than those with poor self-rated health. Sleep efficiency was relatively stable across different seasons and days of the week. The correlation coefficients between device-measured sleep parameters during accelerometer wear and self-reported total sleep duration at baseline assessment were all below 0.25 (Supplementary Fig. 18). The distributions of device-measured overnight sleep duration tend to have a greater variability for participants who self-reported to have less than 5 or greater than 10 h of total sleep duration (Supplementary Fig. 19). Overall, older participants have a shorter REM sleep than younger participants (Supplementary Fig. 20). No major differences were seen between females and males.

**Table 1:** Overall sleep parameters by participant characteristics in the UK Biobank (mean ± SD) for overnight sleep duration, non-rapid-eye-movement sleep (NREM), rapid-eye-movement sleep (REM) and sleep efficiency

Characteristics		Overnight sleep	NREM	REM	Sleep efficiency
	*n* (%)	h/day	h/day	h/day	%
Overall	66,262 (100.0)	6.8 ± 0.9	5.3 ± 0.9	1.5 ± 0.6	81.5 ± 8.6
Age, year					
40–49	6119 (9.2)	6.7 ± 0.9	4.9 ± 0.8	1.8 ± 0.6	81.7 ± 8.2
50–59	20,146 (30.4)	6.7 ± 0.9	5.1 ± 0.9	1.6 ± 0.6	81.1 ± 8.5
60–69	29,216 (44.1)	6.8 ± 0.9	5.4 ± 0.9	1.5 ± 0.6	81.6 ± 8.7
70–79	10,781 (16.3)	6.8 ± 1.0	5.5 ± 1.0	1.3 ± 0.6	82.1 ± 8.8
Sex					
Female	38,552 (58.2)	6.9 ± 0.9	5.3 ± 0.9	1.6 ± 0.6	82.0 ± 8.3
Male	27,710 (41.8)	6.7 ± 1.0	5.2 ± 1.0	1.4 ± 0.6	80.9 ± 9.0
Ethnicity					
Non-white	2004 (3.0)	6.2 ± 1.1	4.7 ± 1.0	1.4 ± 0.6	77.7 ± 10.2
White	64,258 (97.0)	6.8 ± 0.9	5.3 ± 0.9	1.5 ± 0.6	81.7 ± 8.5
Physical activity level					
low < 24.08 mg	22,075 (33.3)	6.9 ± 1.0	5.4 ± 1.0	1.5 ± 0.6	80.8 ± 9.2
Medium 24.08–30.42 mg	22,082 (33.3)	6.8 ± 0.9	5.3 ± 0.9	1.5 ± 0.6	81.6 ± 8.4
High > 30.42 mg	22,105 (33.4)	6.7 ± 0.9	5.1 ± 0.9	1.6 ± 0.6	82.2 ± 8.1
Smoking status					
Never smoker	38,960 (58.8)	6.8 ± 0.9	5.3 ± 0.9	1.5 ± 0.6	81.5 ± 8.5
Ex-smoker	22,884 (34.5)	6.8 ± 0.9	5.3 ± 0.9	1.5 ± 0.6	81.7 ± 8.6
Current smoker	4418 (6.7)	6.7 ± 1.0	5.2 ± 1.0	1.4 ± 0.6	81.2 ± 9.3
Alcohol consumption					
Never drinker	3612 (5.5)	6.6 ± 1.1	5.2 ± 1.0	1.4 ± 0.6	80.7 ± 9.6
<3 times per week	30,099 (45.4)	6.8 ± 0.9	5.3 ± 0.9	1.5 ± 0.6	81.3 ± 8.6
3+ times per week	32,551 (49.1)	6.8 ± 0.9	5.3 ± 0.9	1.5 ± 0.6	81.8 ± 8.5
Education					
School leaver	14,655 (22.1)	6.9 ± 1.0	5.4 ± 0.9	1.5 ± 0.6	81.1 ± 8.9
Further education	21,717 (32.8)	6.8 ± 1.0	5.3 ± 0.9	1.5 ± 0.6	81.3 ± 8.7
Higher education	29,890 (45.1)	6.8 ± 0.9	5.2 ± 0.9	1.5 ± 0.6	81.9 ± 8.3
Townsend Deprivation Index					
Least deprived (<−3.8)	16,559 (25.0)	6.9 ± 0.9	5.3 ± 0.9	1.5 ± 0.6	81.8 ± 8.4
Second least deprived	16,570 (25.0)	6.8 ± 0.9	5.3 ± 0.9	1.5 ± 0.6	81.7 ± 8.4
Second most deprived	16,566 (25.0)	6.8 ± 0.9	5.3 ± 0.9	1.5 ± 0.6	81.5 ± 8.6
(−2.5 to −0.2)					
Most deprived (>−0.2)	16,567 (25.0)	6.7 ± 1.0	5.2 ± 0.9	1.5 ± 0.6	81.1 ± 8.9
BMI					
<18.5, underweight	396 (0.6)	6.9 ± 0.9	5.3 ± 0.9	1.6 ± 0.7	83.1 ± 8.9
18.5–24.9, normal	26,787 (40.4)	6.9 ± 0.9	5.3 ± 0.9	1.6 ± 0.6	82.2 ± 8.2
25–29.9, overweight	26,931 (40.6)	6.8 ± 0.9	5.3 ± 0.9	1.5 ± 0.6	81.3 ± 8.7
30+, obese	12,148 (18.3)	6.6 ± 1.0	5.2 ± 1.0	1.4 ± 0.6	80.5 ± 9.2
Employment					
Employed	41,673 (62.9)	6.7 ± 0.9	5.2 ± 0.9	1.6 ± 0.6	81.4 ± 8.5
Not employed	24,589 (37.1)	6.9 ± 1.0	5.4 ± 1.0	1.4 ± 0.6	81.7 ± 8.8
Self-rated health					
Poor	1281 (1.9)	6.6 ± 1.3	5.3 ± 1.2	1.3 ± 0.6	80.2 ± 10.2
Fair	9168 (13.8)	6.7 ± 1.0	5.3 ± 1.0	1.4 ± 0.6	80.6 ± 9.2
Good	40,146 (60.6)	6.8 ± 0.9	5.3 ± 0.9	1.5 ± 0.6	81.5 ± 8.5
Excellent	15,667 (23.6)	6.8 ± 0.9	5.3 ± 0.9	1.6 ± 0.6	82.2 ± 8.2
Day					
Weekday	66,262 (100.0)	6.7 ± 1.0	5.2 ± 1.0	1.5 ± 0.6	81.7 ± 9.0
Weekend	66,262 (100.0)	7.0 ± 1.2	5.4 ± 1.2	1.6 ± 0.8	81.2 ± 10.5
Wear season					
Spring	14,729 (22.2)	6.8 ± 0.9	5.3 ± 0.9	1.5 ± 0.6	81.7 ± 8.6
Summer	18,211 (27.5)	6.7 ± 0.9	5.2 ± 0.9	1.5 ± 0.6	81.6 ± 8.5
Autumn	18,698 (28.2)	6.8 ± 0.9	5.3 ± 0.9	1.5 ± 0.6	81.5 ± 8.5
Winter	14,624 (22.1)	6.8 ± 1.0	5.3 ± 0.9	1.5 ± 0.6	81.3 ± 8.7

**Fig. 3:**
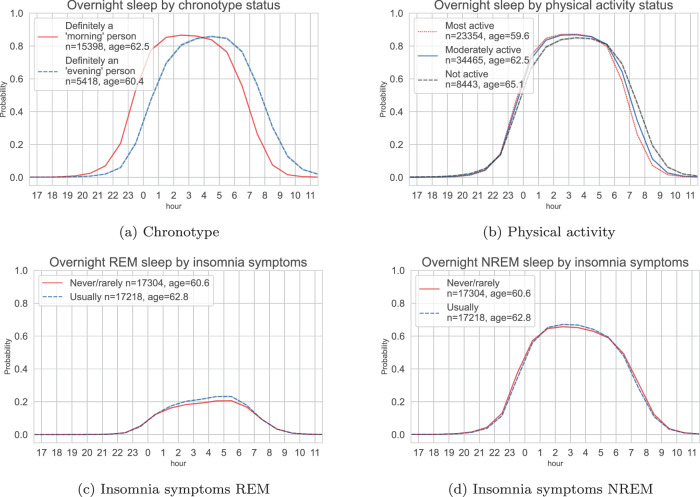
Device-measured sleep probability trajectories throughout the day for the UK Biobank participants. Top: variations of the average overnight sleep probability for the participants with self-reported ‘morning’ and ‘evening’ chronotype (**a**) and the overnight sleep distributions across thirds of device-measured physical activity level (**b**). Bottom: variations of the average REM (**c**) and NREM (**d**) probability in participants with a history of self-reported insomnia symptoms versus those without. REM rapid-eye-movement sleep, NREM non-rapid-eye-movement sleep.

We found expected sleep-wake patterns in population subgroups. For example, timing of the sleep opportunity for participants with a self-reported ‘morning’ chronotype was about 1 h earlier when compared with those that had a self-reported ‘evening’ chronotype (Fig. 3a). We saw similar but shorter phase advance (~30 min) in participants who were most physically active compared to the participants that were least physically active (Fig. 3b). When comparing groups that had a history of self-reported insomnia symptoms versus those who did not, we found that participants with a history of insomnia symptoms were more likely to be in REM sleep on average during the overnight sleep window (Fig. 3c, d). Participants with a history of self-reported insomnia symptoms tended to have a longer overnight sleep duration but with a lower sleep efficiency (Supplementary Fig. 21). The sleep architecture for different population subgroups were similar between weekdays and weekends, with a slight phase delay over the weekend (Supplementary Fig. 22).

### Association with all-cause mortality

**Fig. 4:**
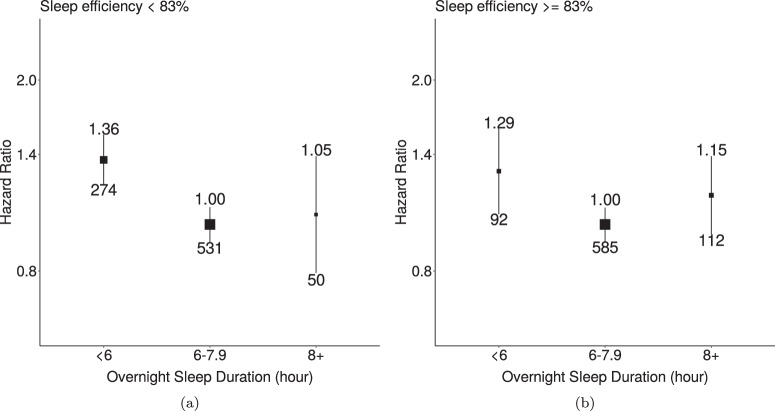
Associations of overnight sleep duration with all-cause mortality for groups with low and high sleep efficiency. **a** participants with less than 85% sleep efficiency; **b** participants with greater or equal to 85% sleep efficiency. The model used 1644 events among 66,262 participants. We used age as the timescale and adjusted for sex, ethnicity, Townsend Deprivation Index of baseline address (split by quarter in the study population), educational qualifications, smoking status, alcohol consumption (Never, <3 times/week, 3+ times/week), overall activity (measured in milli-gravity units). The median was used to separate groups with low and high sleep efficiency. Areas of squares represent the inverse of the variance of the log risk. The I bars denote the 95% confidence interval for the floated risks.

Over 452,652 years of the follow-up, 1644 mortality events among 66,262 participants were observed. Short sleepers (<6 h) had a higher risk of mortality in groups of low sleep efficiency (Hazard ratios (HRs): 1.36; 95% confidence intervals (CIs): 1.18–1.58) and high sleep efficiency (HRs: 1.29; 95% CIs: 1.04–1.61) compared to participants with normal sleep duration (6–7.9 h, Fig. 4). The risk of all-cause mortality appeared to decrease linearly as sleep efficiency increased. However, a non-linear association was observed in the association for overnight sleep duration (Supplementary Fig. 23). When further adjusted for BMI, associations of overnight sleep duration and sleep efficiency with all-cause mortality were slightly attenuated (Supplementary Figs. 24–25). Longer overnight sleep duration was not found to have a higher risk than the reference group (Supplementary Fig. 23).

## Discussion

We have developed, and internally and externally validated a deep learning method to characterise sleep architecture from a wrist-worn accelerometer with competitive performance against 1113 nights of laboratory-based polysomnography recordings. When applying our developed method in the UK Biobank in an epidemiological analysis of 66,214 participants, we found that shorter sleep time was associated with an increased risk of all-cause mortality individually regardless of sleep continuity, indexed by sleep efficiency. Our open-source algorithm and the inferred sleep parameters will open the door to future studies on sleep and sleep architecture using large-scale accelerometer databases.

Our novel self-supervised deep learning sleep staging method outperformed existing baseline methods that rely on hand-crafted features. The inferred sleep architecture estimates had a fair agreement (*κ* = 0.37) with the polysomnography ground truth on the internal validation^12^. Unlike previous work in sleep classification methods that depended on hand-crafted features^13,14^, our proposed method automatically extracted the features using self-supervision, hence removing the need for manual engineering. Even for sleep/wake classification, SleepNet achieved comparable results to a systematic evaluation of eight state-of-the-art sleep algorithms^8^ in the Newcastle dataset. However, our work offers a more robust evaluation and identifies the upper limit of using accelerometry for sleep classification by developing a model with one of the largest multicentre datasets with polysomnography ground truth, at least ten times the size of existing studies.

In the subsequent epidemiological analysis, we found a clear association between short overnight sleep duration with increased risk of all-cause mortality in both good and poor sleepers defined by sleep efficiency. Short overnight sleep duration has been linked with mortality outcomes in self-report and actigraphy-based studies^15,16^. However, few studies have investigated the joint effect of sleep duration and efficiency. One recent study has suggested that participants with short and long total sleep time had an increased risk after accounting for sleep efficiency^17^. However, our analysis did not find that long overnight sleep duration was associated with increased risk, potentially because we did not include daytime naps in our measurement of overnight sleep duration. Daytime napping has been found to be associated with an increased risk of cardiovascular events and deaths in those with longer nighttime sleep^18^. We did not find a U-shape association between device-measured sleep and mortality that has been suggested by other smaller studies^15^. Instead, our data are supportive of adverse associations with short sleep duration only, which is concordant with pre-clinical human and animal studies^19^.

This study has several strengths, including the analysis of sleep architecture in a large, prospective Biobank with longitudinal follow-up. Compared with self-reported sleep questionnaires that only captured sleep duration to the nearest hour, actigraphy-based methods like ours can provide more fine-grained sleep duration and efficiency estimates. The extensive multicentre evaluation of the sleep classification allowed for the characterisation of the measurement uncertainty and a less biased interpretation of the health association analysis. Sleep stage identification from actigraphy is highly challenging, especially for wake periods in bed that are not characterised by wrist movement. With the proposed SleepNet, we could obtain sleep architecture estimates for population health inference after evaluating the face validity of the sleep parameters in the UK Biobank. While future work might improve sleep staging performance by incorporating additional physiological signals, such as electrocardiogram, to improve sleep staging performance, multi-modal sensor signals are not yet available for population-scale studies with longitudinal follow-up beyond a few years^20^. Despite our best efforts to include diverse validation cohorts from different centres, the included datasets mainly consist of healthy populations from a Caucasian ethnic background. Validation in populations with chronic diseases and different ethnic backgrounds would aid in quantifying the measurement uncertainty. Laboratory-based polysomnography is known to suffer from the first-night effect consisting of a reduction in sleep duration, quality and continuity^21^. Future validation studies could also assess the within-person variability using multi-night polysomnography.

In this work, we have developed and validated an open-source sleep staging method that substantially improves the ability to measure sleep characteristics with wrist-worn accelerometers in large biomedical datasets. Using the sleep parameters generated by our model, we demonstrated that shorter overnight sleep was associated with a higher risk of all-cause mortality in both good and poor sleepers. Our proposed method provides the community with a rich set of new measurements to study how sleep parameters are longitudinally associated with clinical outcomes.

## Methods

### Study participants

We used the UK Biobank accelerometry dataset^22^ for two purposes: learning health-relevant accelerometer features to support the training of the sleep staging model and conducting the downstream health association analyses using the developed sleep staging model.

For sleep staging model development, internal validation consisted of two generations of participants from the Raine Study^23,24^ and a sleep patient population from the Newcastle cohort^25^. The Raine Study has followed up roughly 2900 children since 1989 in Australia. A subset of children (Raine Generation 2, Gen2) at the age of 22 and their parents (Raine Generation 1, Gen1) were invited to undergo one night of laboratory-based polysomnography at Western Australia’s Center for Sleep Science. The external validation consisted of two general populations from Leicester^26^ and Pennsylvania^27^. Detailed population characteristics and inclusion criteria are listed in Supplementary Section 1.1.

### Accelerometer devices and data preprocessing

Three different devices were used to collect the accelerometry for the included datasets, ActiGraph GT3X, Axivity AX3 and GENEActive Original accelerometers. The devices used have been shown to have a high inter-instrument agreement (>80%) in derived sedentary and sleep-related time estimates in free-living environments^28^. As for device placement, we selected data from the dominant wrist where possible to be consistent with the UK Biobank protocol.

We used the Biobank Accelerometer Analysis Tool^29,30^ to preprocess all the data. The raw tri-axial accelerometry was first resampled into 30 Hz and clipped to ±3 g. The accelerometry sequence was then divided into consecutive 30-s windows. We considered stationary periods (x/y/z sd < 13 mg) with a duration greater than 60 min as non-wear^22^. We further excluded the data that could not be parsed, had unrealistic high values (>200 mg), or were poorly calibrated.

### Ascertainment of sleep stages via polysomnography

The gold-standard, laboratory-based polysomnography sleep label was aligned with its concurrent accelerometer data as the model ground truth. The polysomnography labels were scored according to the American Academy of Sleep Medicine (AASM) protocol^31^, which divided sleep into five categories: wake, REM and NREM I, II and III. In total, 1,157,913 (~10,000 h) sleep windows were used to train the network. The sleep stage distributions were similar across all the datasets except for the Newcastle cohort, which had a greater proportion of wakefulness than the others (Supplementary Fig. 1).

### Deep learning analysis of sleep stages from wrist-worn accelerometers

A deep recurrent neural network (SleepNet) was trained to classify the sleep stages for every 30-s window of tri-axial accelerometry data. The SleepNet has three components: a ResNet-17 V2^32^ with 1D convolution for feature extraction, a bi-directional Long-Short-Term-Memory (LSTM) network for temporal dependencies learning^33^ and two fully-connected layers for sleep stage prediction. During training, we provided the SleepNet with five-stage polysomnography labels (wake, REM and NREM I, II, III). When evaluating the model, we collapsed all the NREM stages into one class for classification (wake/REM/NREM). Similarly, we collapsed all the REM and NREM stages together to classify wake vs sleep.

The SleepNet was pre-trained using multi-task self-supervision on the UK Biobank to learn features of human motion dynamics^34^. Multi-task self-supervision automatically extracts the features relevant to motion by learning to discriminate different spatiotemporal transformations applied to the unlabelled 700,000 person-days of data. Self-supervised pre-training has been shown to help classify human activity recognition not just in healthy but clinical populations^35^. See Supplementary Section 1.2 for further details of the model development.

For internal validation, we used subject-wise five-fold cross-validation on the Raine Gen2, Raine Gen1 and Newcastle cohorts. For external validation, we trained the SleepNet on all the internal datasets and then evaluated its performance on the Leicester and Pennsylvania cohorts. We compared the SleepNet performance with a random forest model that used the hand-crafted spatiotemporal features^13,30^. The random forest feature definitions are listed in Supplementary Table 2.

We reported the staging performance in both subject-wise and epoch-to-epoch fashion. Three-class and five-class confusion matrices were plotted for both internal and external validation. Since Cohen Kappa, F1 scores and balanced accuracies (Supplementary Table 3) are less influenced by class imbalance, they were used to evaluate the overall model. To assess the relationship between the model performance and population characteristics, we stratified the subject-wise sleep staging performance by age, sex, employment status, income level, body mass index (BMI), presence and severity of sleep apnoea using the apnoea-hypopnea index (AHI), existing sleep disorders and neurological disorders where available.

Finally, we evaluated the agreement between summary sleep parameters per each night derived from our deep learning method and polysomnography via Bland-Altman plots for the following sleep parameters: total sleep duration, sleep efficiency (proportion of total time asleep when in bed), time awake after sleep onset (WASO), REM duration, NREM duration, REM ratio, NREM ratio. Supplementary Table 4 entails the sleep parameter definitions and their calculations.

### Measurements of sleep in 100,000 UK Biobank participants

We obtained the sleep architecture estimates on the UK Biobank by applying SleepNet on the longest overnight sleep windows. Since no concurrent sleep diaries were collected in the UK Biobank, we used a random forest model trained on sleep diaries with Hidden Markov Models smoothing to first obtain time in bed^29,30^. The random forest model achieved 90%+ precision and recall for detecting sleep windows in 152 free-living participants with sleep diaries that asked two questions: ‘What time did you first fall asleep last night?’ and ‘What time did you wake up (eyes open, ready to get up)?’^30^. We used the sleep window output from the random forest model as a proxy for the time in bed. We then merged any time in bed windows within 60 min of one another^36^. Finally, we applied the SleepNet on the longest window over each noon-to-noon interval to estimate the overnight sleep duration. The difference between overnight and total sleep duration is that total sleep duration is a sleep parameter used to assess the agreement between our SleepNet output and polysomnography for model validation. In a single night of polysomnography, the total sleep duration refers to the total time spent in sleep, whereas in a free-living environment, total sleep duration consists of both napping and overnight sleep duration. Overnight sleep duration refers to the estimate for the amount of sleep one obtains for a noon-to-noon interval in a free-living environment using a random forest model for sleep window detection and the SleepNet for sleep stage identification.

We simulated the effects of random missing data on the participants that had no missing data across 7-days to determine the minimum wear time required for stable weekly sleep parameter estimates (Supplementary Section 1.3.2). We found that a minimum of 22 h of wear time per day for at least 3 days were required to ensure the intra-class correlation was greater than 0.75 between the weekly average sleep duration from incomplete and perfect wear data. Moreover, we tried to mitigate the weekend effect by only including the participants who had at least one weekday and one weekend day during the device wear. Shift workers and participants whose data had daylight saving cross-overs were also excluded, as circadian disruption is not the focus of our paper.

Descriptive analyses were performed on the device-measured sleep parameters in the UK Biobank to quantify variations by age, sex, device-measured physical activity level, self-reported chronotype and insomnia symptoms. Estimated marginal means, adjusted for age and sex, were also calculated for different self-rated health groups and self-reported insomnia symptoms.

This research has been conducted using the UK Biobank Resource under Application Number 59070. The UK Biobank received ethical approval from the National Health Service National Research Service (Ref 21/NW/0157). Written informed consent was obtained from all the participants.

### Health association analysis

The associations of overnight sleep duration and sleep efficiency with incident mortality were assessed using Cox proportional hazards regression. All-cause mortality was determined using death registry data (obtained by UK Biobank from NHS Digital for participants in England and Wales and from the NHS Central Register, National Records of Scotland, for participants in Scotland). Participants were censored at the earliest of UK Biobank’s record censoring date for mortality data (2021-09-30 for participants in England and Wales and 2021-10-31 for participants in Scotland, with country assigned based on baseline assessment centre). Cox models used age as the timescale, and the main analysis was adjusted for sex, ethnicity, Townsend Deprivation Index, educational qualifications, smoking status, alcohol consumption and overall activity. See Supplementary Section 1.3.1 for the full specification of the analysis.

### Reporting summary

Further information on research design is available in the Nature Research Reporting Summary linked to this article.

### Supplementary information

Reporting Summary

Supplementary information

## Data Availability

The data for the Newcastle cohort is available from direct download via https://zenodo.org/record/1160410#.Y-O65i-l1qs. The data for other cohorts can be requested by contacting the corresponding host institute.

## Code availability

All the sleep staging models and analysis scripts are freely available for academic use on GitHub: https://github.com/OxWearables/asleep.
