# Author Correction: Self-supervised learning of accelerometer data provides new insights for sleep and its association with mortality

**DOI:** 10.1038/s41746-024-01148-y

**Published:** 2024-07-01

**Authors:** Hang Yuan, Tatiana Plekhanova, Rosemary Walmsley, Amy C. Reynolds, Kathleen J. Maddison, Maja Bucan, Philip Gehrman, Alex Rowlands, David W. Ray, Derrick Bennett, Joanne McVeigh, Leon Straker, Peter Eastwood, Simon D. Kyle, Aiden Doherty

**Affiliations:** 1 Nuffield Department of Population Health, University of Oxford, Oxford, UK; 2 Big Data Institute, Li Ka Shing Centre for Health Information and Discovery, University of Oxford, Oxford, UK; 3 Diabetes Research Centre, University of Leicester, Leicester, UK; 4 College of Medicine and Public Health, Flinders University, Adelaide, Australia; 5 Centre of Sleep Science, School of Human Sciences, University of Western Australia, Crawley, WA Australia; 6 West Australian Sleep Disorders Research Institute, Department of Pulmonary Physiology, Sir Charles Gairdner Hospital, Nedlands, WA Australia; 7 Department of Genetics, University of Pennsylvania, Philadelphia, PA USA; 8 Department of Psychiatry, University of Pennsylvania, Philadelphia, PA USA; 9 NIHR Leicester Biomedical Research Centre, University of Leicester, Leicester, UK; 10 NIHR Oxford Biomedical Research Centre, John Radcliffe Hospital, Oxford, UK; 11 Oxford Centre for Diabetes, Endocrinology and Metabolism, Oxford Kavli Centre for Nanoscience Discovery, University of Oxford, Oxford, UK; 12 Medical Research Council Population Health Research Unit, University of Oxford, Oxford, UK; 13 Curtin School of Allied Health, Curtin University, Perth, WA Australia; 14 Health Futures Institute, Murdoch University, Perth, WA Australia; 15 Sir Jules Thorn Sleep & Circadian Neuroscience Institute, Nuffield Department of Clinical Neurosciences, University of Oxford, Oxford, UK

**Keywords:** Epidemiology, Translational research

Correction to: *npj Digital Medicine* 10.1038/s41746-024-01065-0, published online 20 May 2024

In this Article the authors have corrected a data cleaning issue. Specifically, some devices (older version of Actigraph) used to collect the wrist-worn accelerometer data for the study could enter sleep mode when the movement detected was low. Based on this, they filtered out the data affected by the sleep mode, only considering data without discontinuity in the data stream. However, another study in their group found this criterion insufficient because when the device enters sleep mode, values from the last timestamp are recorded for the entire second to the device, making it a continuous data stream. Therefore, all the data with repeated values for over one second were filtered out. The impact of the issue was limited to 205 out of 1448 nights of training data. After retraining on the clean dataset, preliminary results show that it enhances the reported sleep staging performance. These changes do not affect the core findings of the research. The accompanying open-source software package has also been updated to reflect these changes. The original article has been corrected.

